# Temporary screw epiphysiodesis for correction of ankle valgus in children with multiple hereditary exostoses: a case-control study

**DOI:** 10.1302/2633-1462.710.BJO-2026-0157.R1

**Published:** 2026-10-09

**Authors:** Sarah I. Schmitz, Moritz Broja, Anne Andresen, Alexandra Doerell, Luisa Saegebrecht, Martin Rupprecht, Kiril Mladenov, Alexander S. Spiro

**Affiliations:** 1 Department of Trauma and Orthopaedic Surgery, University Medical Center Hamburg-Eppendorf, Hamburg, Germany; 2 Department of Pediatric Orthopaedics, Altonaer Children´s Hospital, Hamburg, Germany; 3 Department of Pediatric Orthopaedics, Schoen Clinic Hamburg Eilbek, Hamburg, Germany

**Keywords:** Ankle valgus, Multiple hereditary exostosis, Temporary screw epiphysiodesis, Lateral distal tibia ankle (LDTA), epiphysiodesis, valgus deformity, ankles, exostoses, Screw breakage, distal tibia, deformities, lateral distal tibia, osteochondromas

## Abstract

**Aims:**

In patients with multiple hereditary exostosis (MHE), the development of ankle valgus is a common deformity. The aim of this study was to evaluate whether temporary screw epiphysiodesis of the medial distal tibia leads to a correction of the deformity and to provide further information on the timing and duration of the operative treatment, considering sex-specific differences and a concomitant fibular shortening or relevant exostoses.

**Methods:**

The data of 37 MHE patients with a total of 68 ankles, and 35 patients with idiopathic ankle valgus with a total of 56 ankles, were analyzed retrospectively. The lateral distal tibia angle was measured before and after treatment. We also assessed complications, relevant exostoses, and a fibular shortening, as well as sex-specific differences in the correction rate.

**Results:**

Ankle valgus improved in all but one patient of the MHE group with a mean valgus correction of 9.6° (control group: 9.9°). The correction rate was significantly lower in the MHE group than in the control group (0.5° vs. 0.8° per month, p < 0.001), resulting in a significantly higher wearing time in the MHE group (20.12 vs. 14.59 months, p = 0.002). No significant sex-specific differences were found neither in the MHE group nor in the control group. Complications were observed in five ankles of the MHE group (screw breakage during explantation) and none in the control group. Concomitant exostoses or fibular shortening did not affect the correction of ankle valgus.

**Conclusion:**

Temporary screw epiphysiodesis is an effective method for the treatment of ankle valgus in MHE patients. As the correction rate per month is lower in MHE patients, operative treatment should be performed earlier to achieve satisfactory correction.

Cite this article: *Bone Jt Open* 2026;7(10):1288–1294.

## Introduction

Multiple hereditary exostosis (MHE) is a genetic condition characterized by multiple osteochondromas and the development of skeletal deformities such as ankle or knee valgus.^1^ In 90% of the cases, osteochondromas occur as solitary bone tumours, while 10% appear in the context of MHE, an autosomal dominant disease with a prevalence of 1:50.000.^2,3^ In most cases of MHE, mutations in the EXT1 and EXT2 genes on the chromosomes 8 and 11 lead to a loss of function of two glycosyl transferases involved in the heparan sulfate biosynthesis, leading to abnormal bone growth.^4^ Spontaneous mutations of these genes are uncommon (less than 10% of MHE patients).^5^

Exostoses may lead to pain, nerve compression syndromes, and limited mobility of the affected joints in MHE patients.^1^ Furthermore, skeletal deformities like ankle valgus may lead to limited range of motion, activity-related pain, and degenerative osteoarthritis.^6^ Radiologically, ankle valgus is quantified via measurement of the lateral distal tibia angle (LDTA). Ankle valgus deformity occurs in about 50% of all MHE patients.^7^

Treatment of MHE includes conservative and operative measures, i.e. physical therapy, pain management, surgical removal of symptomatic exostoses, and surgical correction of deformities, like knee or ankle valgus.^8^ Multiple surgical techniques exist for the correction of these skeletal deformities. Ankle valgus may be addressed via temporary epiphysiodesis of the medial distal tibia with screws, staples, or plates or, in older patients, correction-osteotomy.^9-11^ The correct timing of operative treatment is important to achieve sufficient correction of ankle valgus. Screw epiphysiodesis for treatment of ankle valgus was first described in 1997; however, the impact of MHE on ankle valgus and its treatment, as opposed to idiopathic ankle valgus, has not been investigated in detail yet.^8,9^ Most studies investigate mainly male patient collectives,^9,10^ possibly because MHE tends to be more severe in males.^12^

Beside an ankle valgus, fibular shortening often occurs in MHE patients. Its extent may be described using the Malhotra classification.^13^ Current data suggest that eight-plate epiphysiodesis does not influence the fibular length,^10^ while several publications discuss the effect of screw epiphysiodesis in patients with MHE, the data on its effect on fibular shortening is limited.^9,14,15^

This retrospective study explores temporary epiphysiodesis of the medial distal tibia in patients with ankle valgus due to MHE. We evaluated whether the forementioned technique led to a correction of the deformity and aimed at providing data for the optimal time and duration of operative therapy in MHE patients, also considering sex-specific differences, relevant exostoses of the distal tibia, and the Malhotra classification.

## Methods

In this study, we retrospectively analyzed the data of all MHE patients treated for ankle valgus via temporary screw epiphysiodesis in our centre (Department of Pediatric Orthopaedics, Altonaer Children's Hospital, Hamburg, Germany) from January 2012 to December 2025. We included 37 MHE patients (11 females and 26 males). Most patients were treated on both ankles, thus a total of 66 ankles were analyzed. As a control group, 35 patients with idiopathic ankle valgus treated via temporary screw epiphysiodesis were included (eight females and 27 males) with a total of 56 ankles. All patients included in this study have completed treatment with screw removal after correction of ankle valgus or reached physeal closure due to completed growth. Ethics approval was obtained from the local ethics committee (Ärztekammer Hamburg; reference numer 2024-300546-WF).

In all cases, the lateral distal tibia angle (LDTA) was measured at implantation and removal of the screws in anterior-posterior radiographs of the lower leg using Zero Footprint (GE Health Care, USA). The LDTA is defined as the angle between the axis of the tibia diaphysis and the distal tibial articular surface. The standard value for the LDTA is 89° ± 3°.^16^ Subsequently, the correction of the LDTA per month was calculated.

The indication for correction of ankle valgus by means of screw epiphysiodesis was: 1) open distal tibial physis; 2) LDTA < 86°; and 3) significant ankle valgus in clinical examination.

The length of the fibula was evaluated using the Malhotra classification: According to Malhotra et al,^13^ fibular shortening can be assessed most accurately by evaluating the relative levels of the distal growth plates of the tibia and fibula (Figure 1). In cases without fibular shortening, the distal fibular growth plate and the talar plateau are on the same level. Mild fibular shortening is defined by the fibular growth plate at the level of the distal tibia epiphysis. In moderate shortening, both distal growth plates are at the same level, whereas in severe shortening, the fibular growth plate is proximal to the tibial growth plate. In addition to the relative assessment of the growth plates, Malhotra et al^13^ describe the wedging of the distal tibial epiphysis (grades 0 to 3).

**Fig. 1:**
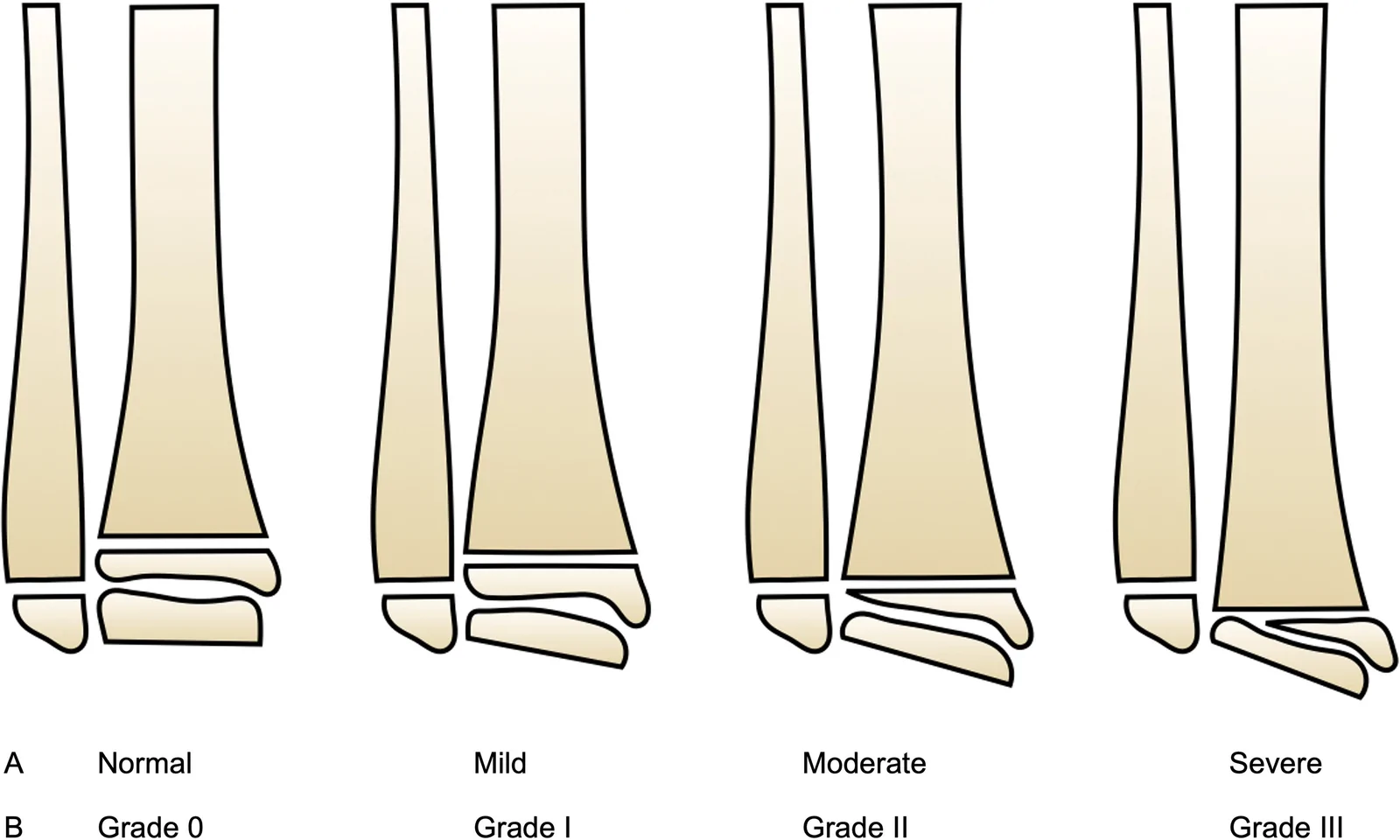
The length of the fibula is graded from normal to severe (a) depending on the relative levels of the distal fibular and tibial growth plate. Additionally, the amount of wedging of the distal tibial epiphysis is graded from grade 0 to III (b). (Figure modified according to Malhotra et al).^13^

We also analyzed whether there was an exostosis at the distal tibia or a synostosis between the distal tibia and fibula (Figure 2). According to Bessler at al,^17^ kissing exostoses (i.e. osteochondromas on two different bones facing each other) are precursors of synostoses in patients with multiple cartilaginous exostoses.^17^ Only significant exostoses that extended to the fibula in the radiograph and synostoses were included in the analysis. Apart from that, complications such as screw breakage were investigated.

**Fig. 2:**
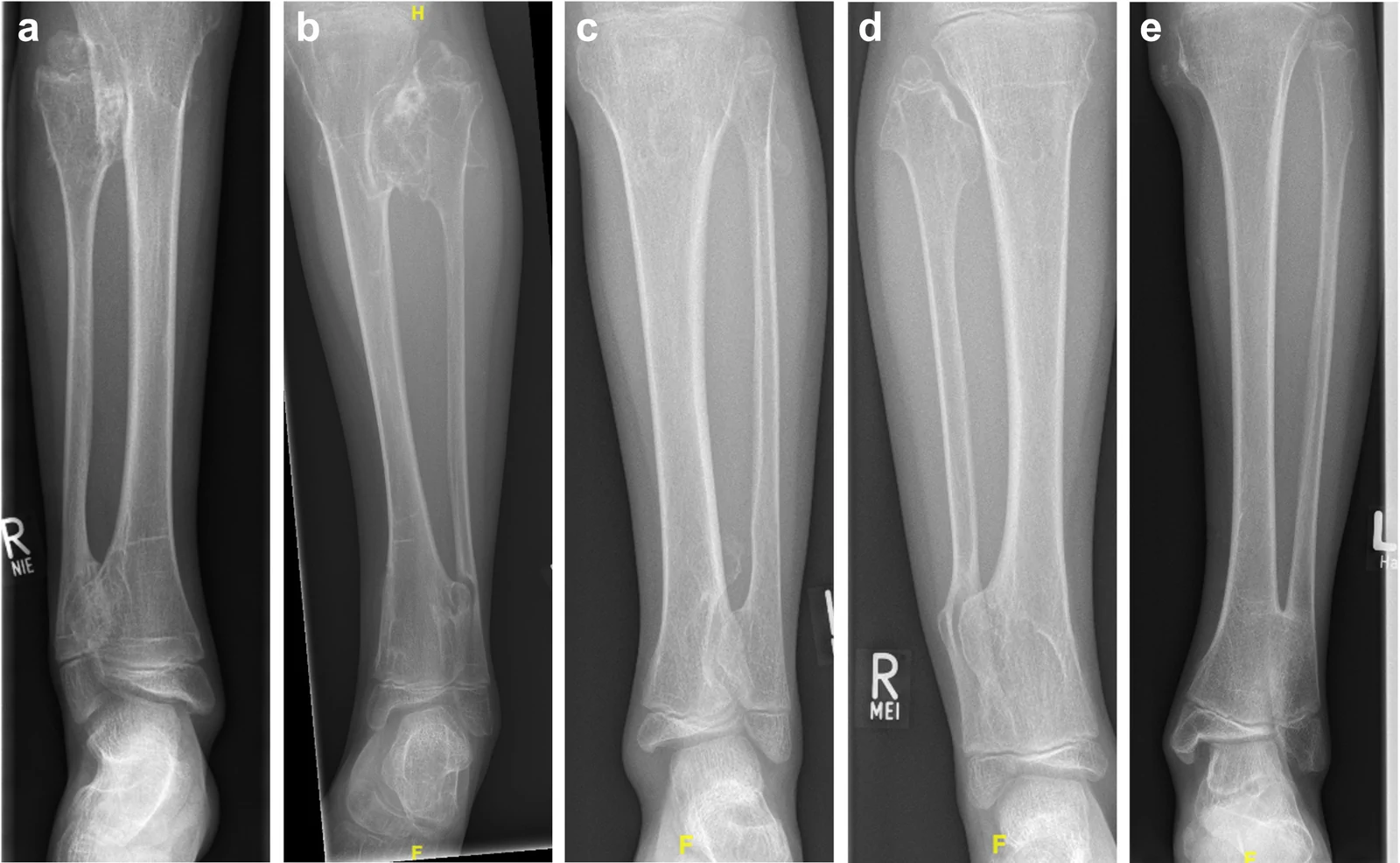
Examples of a relevant exostosis (a to c) or synostosis (d,e) between the distal tibia and fibula in anteroposterior X-rays of the lower limb.

Temporary screw epiphysiodesis of the medial distal tibia was performed using a cannulated 4.5 mm fully threaded titanium screw under general anesthesia with the patient in supine position on a radiolucent table (Figure 3). An incision of about 1 cm is made parallel to the skin tension lines distal of the medial malleolus. The tendon of the tibialis posterior muscle is held aside, and preparation is continued to the bone. Then, a Kirschner wire is placed into the medial malleolus and its location is controlled in the anteroposterior (AP) and lateral view. After monocortical overdrilling and measuring of the screw length, the screw is placed into the medial malleolus. Its position is again controlled in the a.p. and lateral view before closure of the wound. The patients were allowed pain-adapted full weightbearing. Clinical and radiological controls were performed every four to six months. Screw removal was performed after full correction of ankle valgus (LDTA 86° to 92°) or physeal closure in both groups or after slight overcorrection (LDTA > 92°) in young MHE patients with further growth potential of the distal tibial physis.

**Fig. 3:**
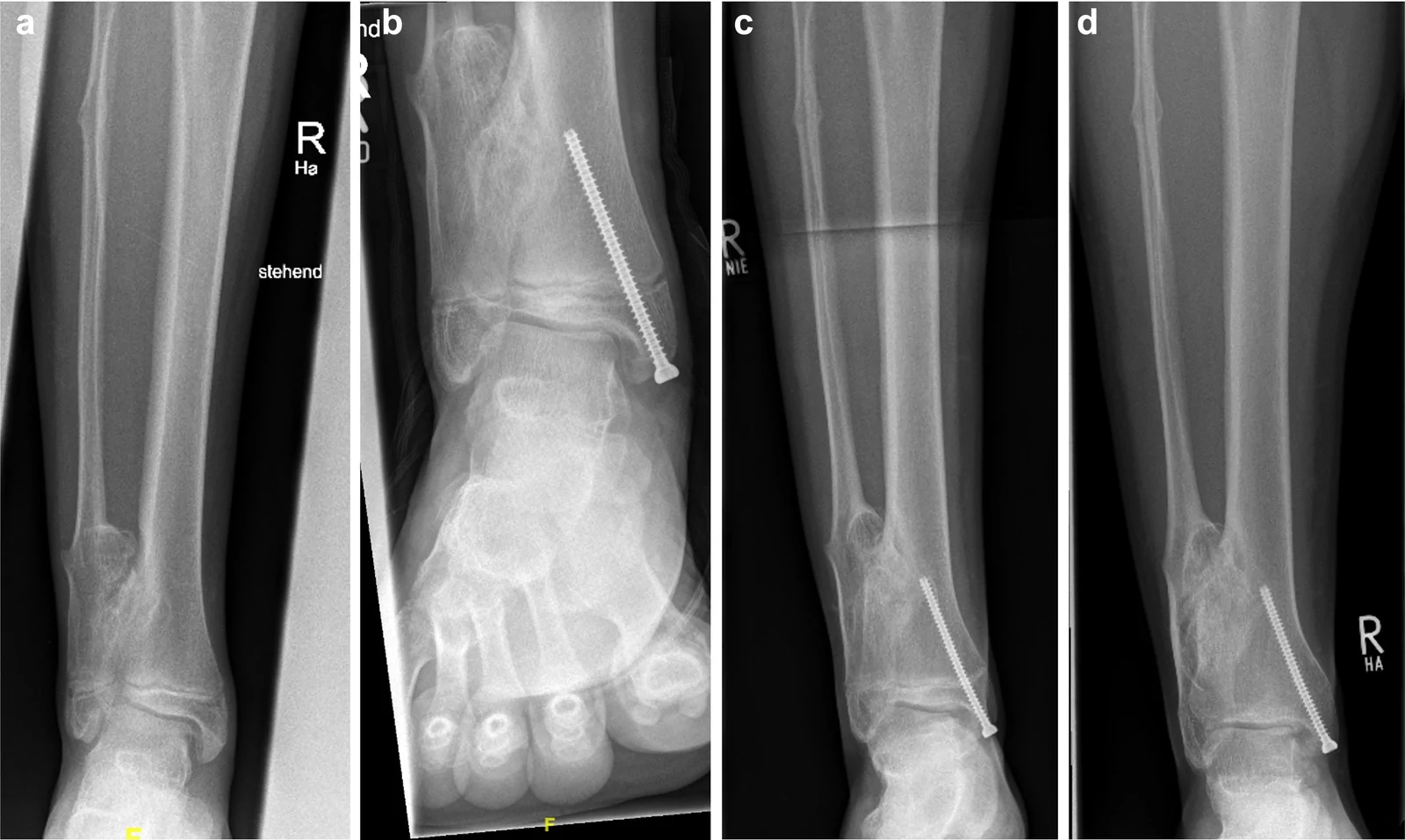
Temporary epiphysiodesis was performed using a cannulated 4.5 mm screw (male patient, age at implantation 13.6 years). The anteroposterior X-rays of the ankle (b) and the lower limb (a, c, d) show the ankle valgus before implantation of the screw (a), as well as the radiological controls after implantation (b) and at follow-up after 11 and 28 months (c, d).

### Statisical analysis

Statistical analysis was performed using R software v. 4.5.2 (R Foundation for Statistical Computing, Austria) including the lme4 and corrplot packages. For baseline variables, mean and standard deviation were obtained for the subgroups. Group comparisons were conducted using linear mixed-effects models implemented in lme4 (lmer function). The fixed effect was group (MHE vs control), with random intercepts by pseudonymous subject ID to account for within-subject dependencies as some patients underwent surgery on both ankles, significance level was set at p < 0.05. Correlations between correction rate and age at implantation or sex were analyzed separately within each group using linear mixed-effects models with correction rate as the outcome using the lme4 and corrplot packages. Pseudonymous subject ID was included as a random intercept to account for non-independence of bilateral measurements within individuals. The significance level was set at p < 0.05.

## Results

At the time of final analysis, all patients have completed treatment, either by screw removal or physeal closure. Mean age at implantation was lower in MHE patients than in the controls (12.0 vs 12.2 years, p = 0.422) while mean age at explantation was higher in MHE patients (13.7 vs 13.6 years, p = 0.987). These differences were however not statistically significant (Table I). As MHE patients underwent implantation earlier and removal later, their wearing time was significantly longer (20.12 vs 14.59 months, p = 0.002; Table I).

**Table I.** Age at implantation and explantation in years of the screw for temporary epiphysiodesis and wearing time in months in both groups.

Group	n	Mean age at implantation, yrs (SD)	Mean age at explantation, yrs (SD)	Mean wearing time, mnths (SD)
MHE	68	12.0 (1.3)	13.7 (1.4)	20.12 (9.38)*
Control	56	12.2 (1.6)	13.6 (1.9)	14.59 (6.53)*

* Significant difference between the multiple hereditary exostosis (MHE) group and the control group.

Ankle valgus improved in all but one MHE patient and in all controls. At implantation, the mean LDTA was 79.6° (SD 4.1°) in MHE patients and 80.2 ° (SD 2.6°) in controls; at explantation, 89.1° (SD 4.8°), and 90.0° (SD 2.9°), respectively. The mean valgus correction was 9.6 (SD 5.1°) in MHE patients and 9.9 (SD 3.9°) in controls. In the MHE group, full correction in a range of 86° to 92° was achieved in 48 out of 68 ankles, whereas overcorrection and undercorrection were observed in ten ankles each. In the control group, 46 of 56 ankles achieved full correction, eight were overcorrected, and two were undercorrected. Overcorrection was intended in patients with relevant remaining growth potential to prevent recurrence of ankle valgus.

The correction rate of the LDTA was found to be significantly lower in the MHE group with 0.51° per month, as opposed to 0.77° per month in the control group (p < 0.001; Table II). The distal physis was closed in 26 cases with MHE and 14 cases of the control group at the time of screw removal. No significant correlation between age at implantation and correction rate per month could be found in either group (MHE group: p = 0.327, control group: p = 0.740). When comparing the correction rate per month of female versus male patients in both groups, sex was not significantly associated with correction rate in either group (MHE group, p = 0.853; control group, p = 0.565).

**Table II.** Correction rate per month of the lateral distal tibia angle (LTDA) in the multiple hereditary exostosis (MHE) group and in the control group in degrees per month.

Group	n	Mean (SD)
MHE	68	0.51 (0.28)*
Control	56	0.77 (0.34)*

* Significant difference between the multiple multiple hereditary exostosis (MHE) group and the control group.

Relevant exostoses or synostoses between the distal tibia and fibula were observed in 49 limbs of the MHE group and none of the controls. Ankle valgus improved after screw epiphysiodesis in all but one patient with exostoses or synostoses: 36 of 49 ankles achieved complete correction, while seven were undercorrected and 6 were overcorrected. In the MHE group, screw breakage during explantation occurred in five ankles. No other complications were found. In four out of these five ankles, we noted a concomitant presence of an exostosis or synostosis. No complications were observed in the controls. In the MHE group, a mild to severe fibular shortening according to the Malhotra classification was found in 60 out of 68 ankles. In 52/60 of the patients (86.7%) with fibular shortening, its grade remained unchanged after temporary screw epiphysiodesis.

## Discussion

In children, valgus deformity of the ankle can be corrected by means of temporary epiphysiodesis using various operation techniques. The use of staples and eight-plates has been discussed in literature.^18,19^ In 1997, the use of 4.5 mm vertical screws for treatment of ankle valgus was described as a safe and effective alternative technique.^8^ Temporary screw epiphysiodesis was further investigated by Davids et al,^20^ who agreed on the technique being effective and associated with low morbidity.^20^

To date, the data on epiphysiodesis of the distal tibia in MHE patients is limited. Most studies include a small number of patients, possibly due to the low prevalence of the disease.^8-10,14^ Additionally, not all studies describe the effects of screw epiphysiodesis as other implants, such as eight-plates or staples, can also be used. Therefore, further investigation of the treatment of ankle valgus in MHE patients is needed to allow correct timing of the surgical intervention, as well as to guide the implant choice. To our knowledge, this is the largest study focusing on screw epiphysiodesis for the correction of ankle valgus in MHE patients in comparison to a control group. We found screw epiphysiodesis to be an effective method in this patient collective, as it led to an improvement of the LDTA of 9.6° on average in all but one case. In this female MHE patient, screw epiphysiodesis was performed at aged 13.4 years with the distal tibial physis nearly closed upon implantation. Complete clinical and radiological correction of the deformity (full correction or overcorrection) was achieved in 58/68 of the MHE ankles (85.3%) after screw epiphysiodesis. According to our data, the correction per month is lower in MHE patients as compared to the control group (0.51° vs 0.77°). This could be associated with common findings in MHE patients such as growth retardation and short stature.^21,22^ Earlier studies on temporary epiphysiodesis of the ankle in MHE patients found comparable correction rates: 0.59° per month^20^ and 0.58° per month.^9^ Additional studies comparing temporary epiphysiodesis in MHE patients to a control group^14,23^ reported lower correction rates in MHE, however, these differences were not statistically significant.^14,23^ In contrast to our study, Driscoll et al^23^ used either screws or eight-plates for epiphysiodesis and included a heterogenous control group, while Dussa et al^14^ investigated five cohorts with different diagnoses, including idiopathic ankle valgus and ICP.

Ozaki et al^24^ investigated the effect of isolated excision of distal osteochondromas on valgus deformity and found that there was none. The effect of these osteochondromas on the operative correction of ankle valgus has, however, not been assessed in earlier studies. Most of our MHE patients had relevant exostoses or a synostosis between the distal tibia and fibula (49/68, 72%). These osteochondromas seem to have no significant impact on the correction of ankle valgus by screw epiphysiodesis, as 42/49 of MHE ankles (86%) with relevant exostoses had full correction or overcorrection in our study.

We found a tendency towards younger age upon implantation in MHE patients compared to the control group. We assume that these differences result from the earlier onset of the deformity in MHE patients and the unpredictability of the expected growth.

According to the available literature, no changes in Malhotra classification can be observed after correction of ankle valgus by means of temporary epiphysiodesis.^9,10,13,25^ Our results confirmed these observations: In 52/60 of the MHE patients (86.7%) with fibular shortening, its amount remained unchanged during follow-up. We observed satisfactory radiological and especially clinical correction of ankle valgus in patients with substantial fibular shortening. Temporary screw epiphysiodesis may thus be considered a suitable, minimally invasive method for this patient collective.

In our cohort, no complications occurred in the control group, while five complications were observed in the MHE group (breakage of the screw during explantation). Interestingly, four out of these five patients had a relevant exostosis or a synostosis between the distal tibia and fibula. Due to the small number of patients and especially complications, a correlation between synostoses and screw breakage cannot be statistically proven. Further studies might investigate a possible correlation between relevant exostoses and synostoses with concomitant complications. Screw breakage did not affect deformity correction during follow-up. In patients with open physes, the fragment blocking the physis was removed by overdrilling under radiological control and epiphysiodesis trough drilling was prevented using bone wax. MHE patients and their parents should be informed about the possible screw breakage and its implications preoperatively. The screws bend during correction of ankle valgus, which complicates their explantation, especially in the case of long screws. We therefore suggest that shorter screws reduce the complication rate. Since 2024, we have thus used shorter screws crossing the physis at approximately 50% of the screw length achieving satisfactory correction of ankle valgus as well.

Correct timing of temporary epiphysiodesis is crucial to achieve full deformity correction. The correction rate has been shown to be inversely proportional to age at implantation in these patients^14^ and MHE patients show a lower correction rate according to our data. Thus, implantation needs to be performed at an earlier age in these children, especially in cases with severe deformity.

Interestingly, we found no sex-specific differences in the correction rate of ankle valgus in both the MHE group and the control group. However, these findings should be validated in a larger cohort, as 47/68 of the ankles (69%) observed in our patient collective were male in the MHE group and 43/56 (77%) in the control group, which is one of the limitations of this study. Interestingly, previous studies reported comparable sex distribution.^9,10^ Another limitation is the retrospective design. As MHE is a rare genetic condition, a multicentric prospective study might be suited to provide sufficiently high sample sizes for all groups.

In conclusion, temporary screw epiphysiodesis of the distal tibia is an effective method to treat ankle valgus in patients with and without MHE. The operation technique is simple with a short operation time, cost-effective, and has low morbidity. It should, however, be considered that the correction rate of ankle valgus is lower in MHE patients, and operative treatment should thus be performed earlier in this patient collective. In our centre, we perform surgery in patients aged ten to 12 years to achieve full correction until physeal closure in MHE patients. Shorter screws should be used to avoid screw breakage but should be long enough to ensure sufficient correction of ankle valgus.

Further studies are necessary to validate our findings on a larger scale, especially concerning the sex-specific differences. Additionally, a prospective multicentric trial investigating the advantages and disadvantages of the existent operative treatment options for ankle valgus in MHE patients would be interesting.

**Take home message**

- Temporary screw epiphysiodesis is an effective method for the treatment of ankle valgus in multiple hereditary exostosis (MHE) patients.

- As the correction rate per month is lower in MHE patients, operative treatment should be performed earlier (at aged ten to 12 years) to achieve satisfactory correction.

## Data Availability

The data that support the findings for this study are available to other researchers from the corresponding author upon reasonable request.
